# Supplementary material for: Protein Phosphatase Ppz1 Is Not Regulated by a Hal3-Like Protein in Plant Pathogen Ustilago maydis
Source: Int J Mol Sci. 2019 Aug 5;20(15):3817. doi: 10.3390/ijms20153817 (PMC6695811; doi:10.3390/ijms20153817)
Supplement: Supplementary file 1 [file ijms-20-03817-s001.pdf]

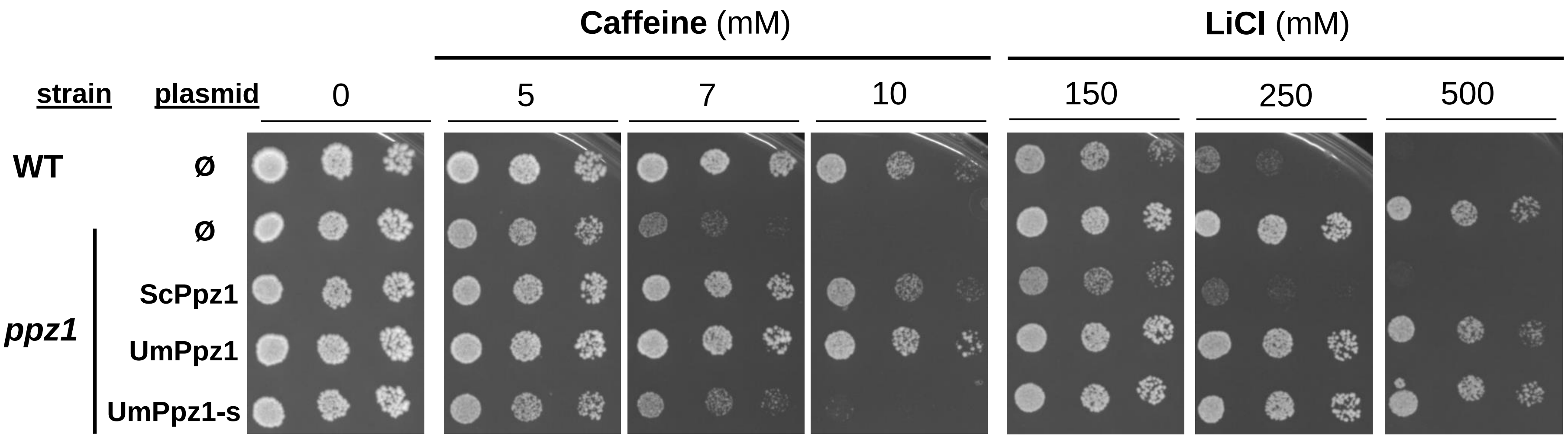

Supplementary Figure S1. Wild type (BY4741) and its *ppz1* derivative were transformed with the indicated plasmids (YEp181-based) expressing a C-terminally HA-tagged version of the indicated proteins . Cells were spotted on the indicated SD (lacking leucine) plates and grown for four days.

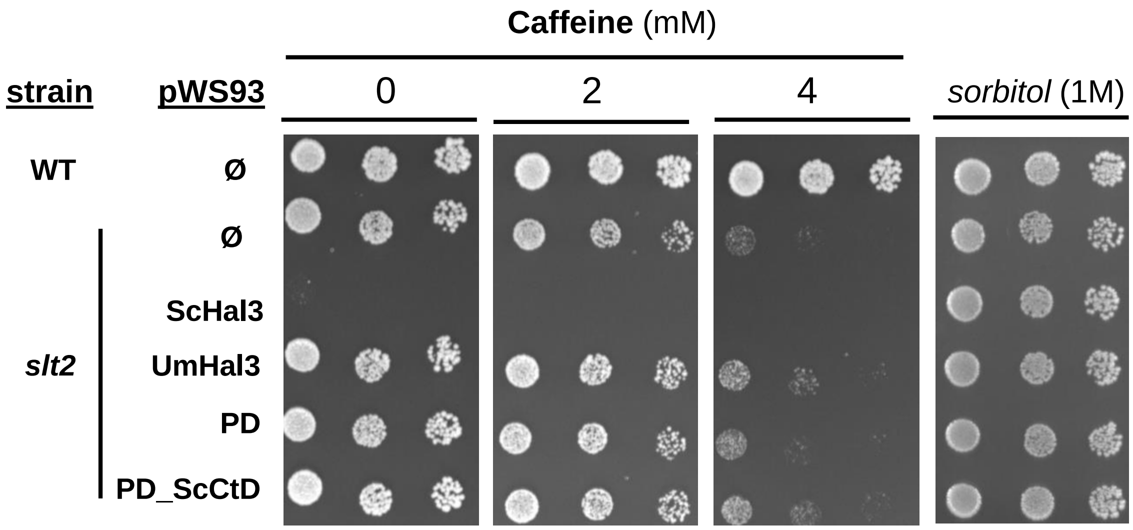

Supplementary Figure S2. Expression of UmHal3 variants in the *S. cerevisiae slt2* background. Cells were spotted on the indicated SD (lacking uracil) plates and grown for 3 days. PD, UmHal3_PD; PD_ScCtD, UmHal3_PD_ScCter.

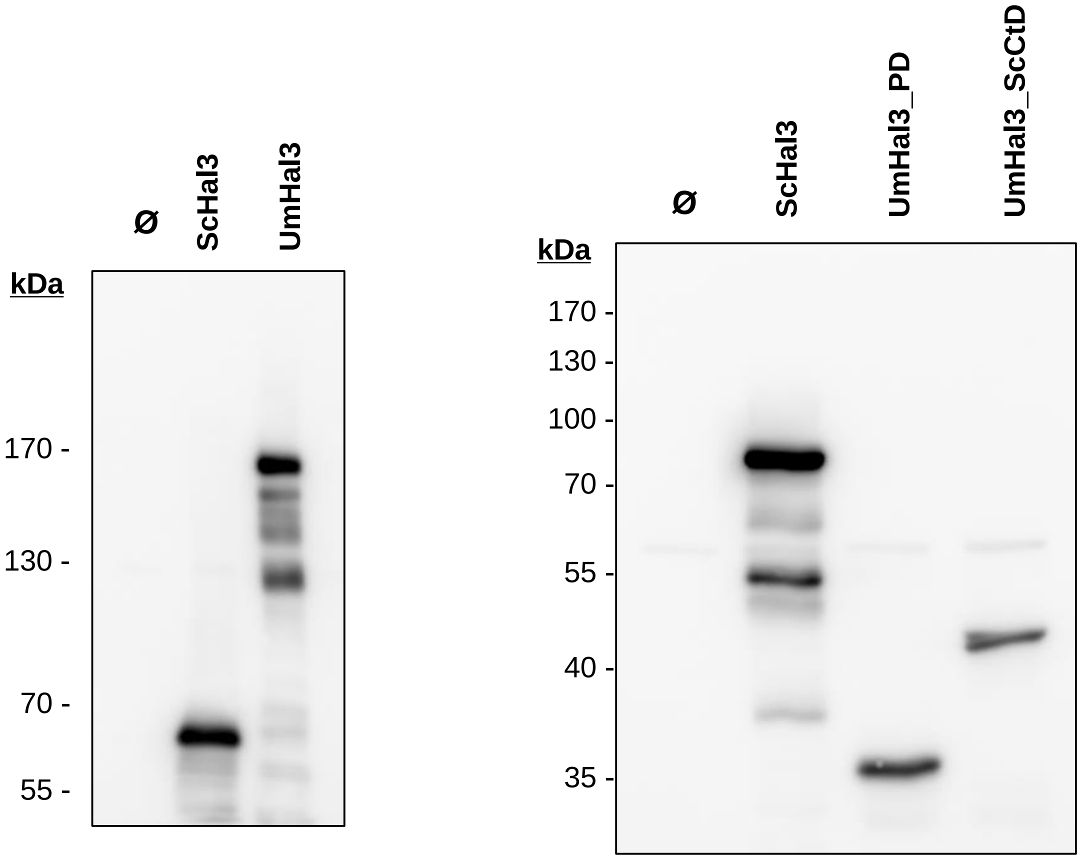

Supplementary Figure S3. Immunoblot analysis of protein extracts of IM21 cells expressing HA-tagged versions of the diverse ScHal3 and UmHal3 proteins used in the binding assays. Forty µg of protein extracts were run on 6% (left panel) or 10% (right panel) SDS-PAGE and transferred to membranes for immunoblot analysis using anti-HA antibodies as indicated in Materials & Methods. Signals were integrated and normalized in order to calculate the amount of extract to be used in the binding assays to ensure equivalent amounts of prey proteins.

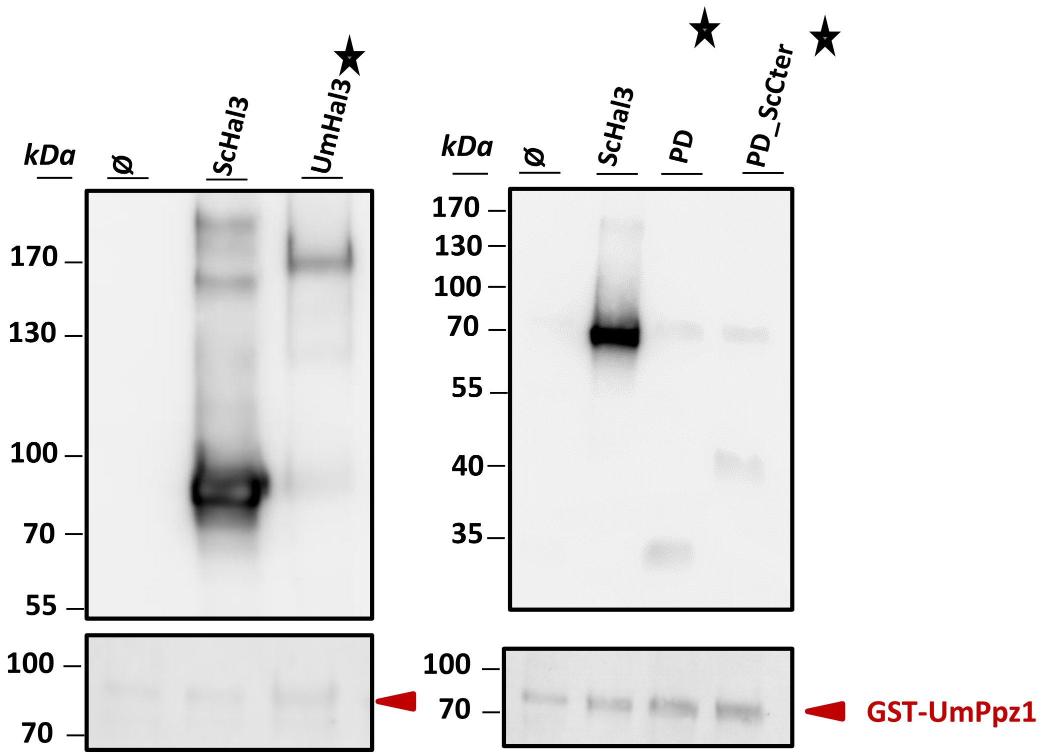

Supplementary Figure S4. The *in vitro* binding experiment was as in Figure 5A, except that beads containing 8 μg of recombinant GST-UmPpz1 were employed and that, in the lanes marked with an asterisk, 50 μl of the samples (instead of 25 μl) were loaded in the gel.

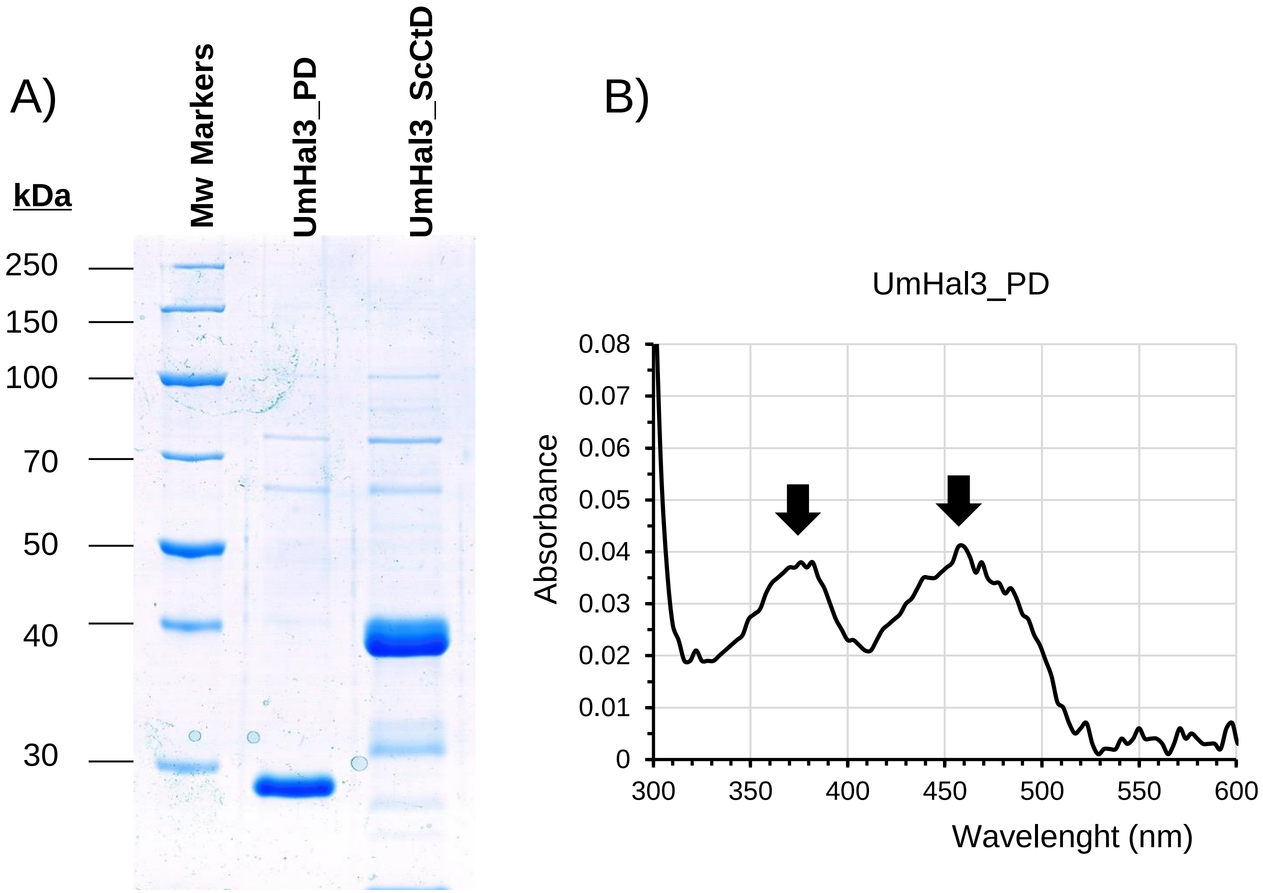

Supplementary Figure S5. A) Coomassie blue staining of a SDS-PAGE analysis of recombinant Umhal3_PD and UmHal3_PD_ScCtD after digestion with PreScission protease. B) UV-visible scan of recombinant UmHal3_PD showing the two peaks characteristics of an oxidized flavin.

A)

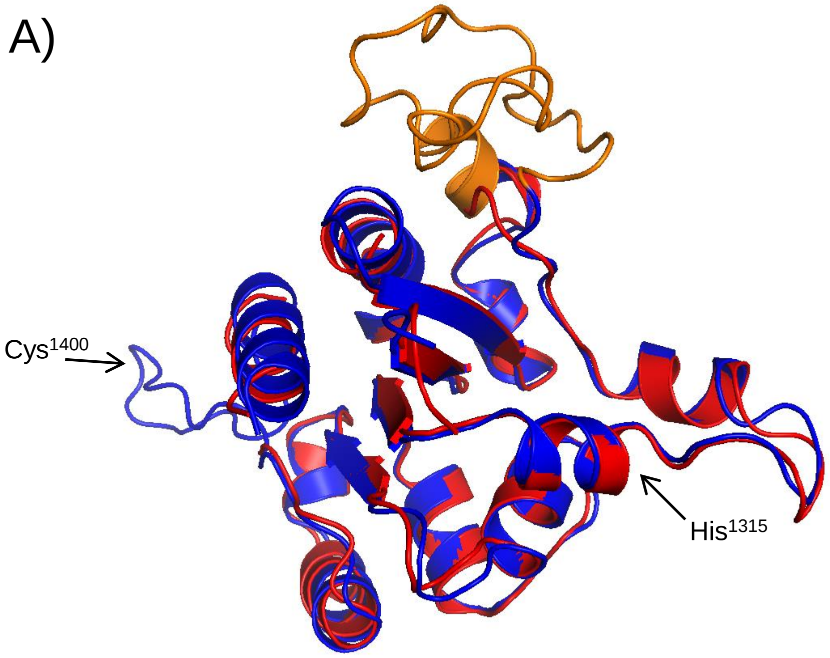

B)

```
UmHal3       KTTRPVQNRTTSYLASQLSRTTATPPRTSSGSLSAVLADEVVAAAKPLPASLRSPYAPLS  1200
Hs_PPCDC     ----------------------------------------------MEPK----ASCPA  9
AtHal3a      ----------------------------------------------MENG--KRDRQDM  11
                                                           :

UmHal3       RPPTCDRPLHIVLASTGSVASVKIPLIVQELLTYANVRVQVIATDNSLHFYDRADIAKLN  1260
Hs_PPCDC     AAPLMERKFHVLVGVTGSVAALKLPLLVSKLLDIPGLEVAVVTTERAKHFYSPQDI----  65
AtHal3a      EVNTTPRKPRVLLAASGSVAAIKFGNLCHCFTEWAE--VRAVVTKSSLHFLDKLSLPQ--  67
                   *   ::::. :****::*:  :    :        *  .:.*. : **  .   .:

UmHal3       AASGGDGDEYTVASLAAENQSASVCGRAASHVRAHLWTNADEWTSFSRIGDPILHIELRR  1320
Hs_PPCDC     ---------------------------------PVTLYSDADEWEMWKSRSDPVLHIDLRR  93
AtHal3a      ---------------------------------EVTLYTDEDEWSSWNKIGDPVLHIELRR  95
                                              . *::: ***   :.   .**:***:***

UmHal3       WADMVLIAPCSANTLAKIYGGMCDDLLTSFVRALARDTPKWMFPAMNTLMWENEVTEVHV  1380
Hs_PPCDC     WADLLLVAPLDANTLGKVASGICDNLLTCVMRAWDRSKPLLFCPAMNTAMWEHPITAQQV  153
AtHal3a      WADVLVIAPLSANTLGKIAGGLCDNLLTCIIRAWDYTKPLFVAPAMNTLMWNNPFTERHL  155
             ***:::::** .****.*: .*:**:***..:**     .*   .  ***** **:: .*  ::

UmHal3       DALRRRGWVVHGPVEKMLACGDMGTGAMVEWTELVQTLV--QWAHLVRDESR---      1430
Hs_PPCDC     DQLKAFGYVEIPCVAKKLVCGDEGLGAMAEVGTIVDKVKEVLFQHSGFQQS----      204
AtHal3a      LSLDELGITLIPPIKKRLACGDYGNGAMAEPSLIYSTVRLF-WESQAHQQTGGTS      209
               *   * .     : * *.*** * ***.*    : ..:    :     :::
```

Supplementary Figure S6. The sequence comprised between residues 1121 to the C-terminus of UmHal3 was submitted to the SWISS-MODEL workspace and a 3D-model generated using the structure of *C. neoformans* Hal3b (PDB ID: 6eoa). The model (deep blue) was aligned with the 3D structure of human PPCDC (PDB ID: 1QZU, red). The catalytic Cys and His are denoted by arows and the insert described in the text is denoted in deep orange. B) Alignment of the region relevant to the structure in panel A for UmHal3, human PPCDC (Hs_PPCDC) and *A. thaliana* Hal3 (AHal3a). Catalytic regions/residues are in color.

Supplementary Figure S7. Protein sequence of UmHal3 and multiple sequence alignment (Clustal Ω 1.2.4) with ScHal3 and ScCab3. The PD domain is underlined and the residues critical for PPCDC activity are denoted in color.

```
UmHAL3      MQRTPSDSLDPSIAASKALDASQSGSDCSSRTTSPRFPIARLEPTDRAEWQSQTVGRSSH      60
ScHal3      ------------------------------------------------------------      0
ScCab3      ------------------------------------------------------------      0

UmHAL3      RRDSQHPFGDHTAQRIVPTSSSTPSTPTSALPTSRTYHSLNYAPRQRNSMLRPNAASVFQ      120
ScHal3      ------------------------------------------------------------      0
ScCab3      ------------------------------------------------------------      0

UmHAL3      PHSPQSAQQQQAVTTAFTGLALPSGRVSPALPSYSSPLSATSYLRTSPSYPTISLPDAVP      180
ScHal3      ------------------------------------------------------------      0
ScCab3      ------------------------------------------------------------      0

UmHAL3      PRDSSRVRDSVSLQADTAPTSLARMQLQQMQDEARRLGLNEKSAGWLILEALQAATEGEW      240
ScHal3      ------------------------------------------------------------      0
ScCab3      ------------------------------------------------------------      0

UmHAL3      AAVAELLANGDATLLLPRDPPSVFTSSSQLSASFAYDHTIFNAASASSTKPSPSAVPSSA      300
ScHal3      ------------------------------------------------------------      0
ScCab3      ------------------------------------------------------------      0

UmHAL3      TCPDALSILTLSGLRGSLSRAPSDQSEPDTLVQAVESQQFQLILQSFVVKTSQSAISNLK      360
ScHal3      ------------------------------------------------------------      0
ScCab3      ------------------------------------------------------------      0

UmHAL3      DASSRQETLEMLAPLPIITSLDKSNCRYPSFSLLASNAQFPLPPPLNVRKASSQSQAGGK      420
ScHal3      ------------------------------------------------------------      0
ScCab3      ------------------------------------------------------------      0

UmHAL3      NRARSNSKLNAAGSRASASFASIFGGSGRERRRQAEAAGTTGSEMLDAQRLSVNATHLDV      480
ScHal3      ------------------------------------------------------------      0
ScCab3      ------------------------------------------------------------      0

UmHAL3      QPFQGANVAQDGLEIESGAQIPSSLGVELRGDLQHTTNDEATSIKESTCATTQRRSVSVW      540
ScHal3      ------------------------------------------------------------      0
ScCab3      ------------------------------------------------------------      0

UmHAL3      VVDHLVRRSTVMKMIRKALDTRIKERLLAKSIPESISDVVASFAATYLPPAPSLNDVSAS      600
ScHal3      ------------------------------------------------------------      0
ScCab3      ------------------------------------------------------------      0

UmHAL3      RSGGAERGQASHSNRSRAASPVNVVNPPYLADPDELSENFQDFFNSIREQLYNFDSSQAE      660
ScHal3      ------------------------------------------------------------      0
ScCab3      ------------------------------------------------------------      0

UmHAL3      YLRADSDTAAAIARNPAPNASLEEQHAQHDRIERQLEAVETVLCEEVYDRIFCPVTSRDR      720
ScHal3      ------------------------------------------------------------      0
ScCab3      ------------------------------------------------------------      0

UmHAL3      YHDDALASRIAALNVLGLSLRHLGLVVPSERLQEDEIEATESNALLDGIERIVQQCGDEL      780
ScHal3      ------------------------------------------------------------      0
ScCab3      ------------------------------------------------------------      0
```

```
UmHAL3      QRLESAMCRSPQAKLDVLVRAHKIAVEGVAQLPSIKMRDDDTAVEGQEKEQTARPSSGKK      840
ScHal3      ------------------------------------MT-------------AVASTSGKQ      11
ScCab3      ------------------------------------MTDE--------KVNSDQNMNGKQ      16
                                                *              :    .**:

UmHAL3      GDGSTSA--DLILPILIYSIVSS-NPSHLASNLLYIQRFR--AESLVQGETSYCLVNVQA      895
ScHal3      DADHN--------------------------------------------QSIECPRFSRG      27
ScCab3      GVNLISSLPTTQVPVSILTNKERRKSIHDESNFERSDSHEDQSKSNSNRRNIYKNDYSTN      76
            . .                                              ..

UmHAL3      AVAFL-ENV---DVKDLGLDSNQIGAHLPVHSRDEGHAPRSSTGSSHSLVPSSKIAETSS      951
ScHal3      QKEILLDH------EDAKGKDSIINSPV---------------SGRQSISPTLSNATTTT      66
ScCab3      LRDFSFANLKQNSERNKDGHEIQINTSMPA----------NTNGQQKRFSPSLPSAVS--      124
               :   :      .:   ..  *.: :               .  : : *:   * :

UmHAL3      ATLAMPARIRGRLTQEIGDLAGASNKVIT-----GVMGSSISAFSRMMGANASVAEAGVD      1006
ScHal3      -------------TKSIMNATGTSGAVVSNTPEPGLKRVPAVTFSDL-------KQQQKQ      106
ScCab3      ------------------------------FTVPEVERLPYHRYSISNKP---GKQQQQQ      151
                                               :       :*          :   :

UmHAL3      ESLGRKRAKSSASLNAAHSEGAVAPTQADAQRPLRSRTSSSVTPTDDSYLGEQSK-EKEK      1065
ScHal3      DSLTQ---------LKNDSERTKSPN--SNP-A---PVSNSI-PGNHAVIPNHTNTSRTT      150
ScCab3      EQLQQ---------NQQQEEQQKAQLQEQNQRA---KQQEEV-K----QIQEQV---QKK      191
            :.* :            ..*   :    .         ...:       : ::    : .

UmHAL3      DTVGTPSTDK-----PSIG-DRLAMLSRLGTSALSSSPSSSLGSGLPSDS---GLGVSAS      1116
ScHal3      QLSGSPLVNEMKDYDPKKKDSALKIVDTMK-------PDKIMATSTPISR----------      193
ScCab3      QTERQQLIDEKE-----------RIANAIF-------KENTTNDGTDIRKHSVSSGTSNS      233
            :       ::              : . :         ..    .

UmHAL3      GQSLIASPR----AVSRELPGPPPPTKTKTTRPVQNRTTSYLASQLSRTTATPPRTSSGS      1172
ScHal3      ----------ENNKVTAKAPTSITLRKEDAQDQANNVS-----GQIN-VRSTPEE-----      232
ScCab3      -EDEVDSPSMEKNSIV-HMPGDFIYFN-----PKSNAS-----KPIT-AKAAPLSA---N      277
                          :  . *      :       .* :       :. . ::*

UmHAL3      LSAVLADEVVAAAKPLPASLRSPYAPLSRPPTCDRPLHIVLASTGSVASVKIPLIVQELL      1232
ScHal3      --TPVKQSVIPS--IIPKRENSKNLD-PRLPQDDGKLHVLFGATGSLSVFKIKPMIKKLE      287
ScCab3      NSTHKNKEVIT----APTGPRVPFTE-FFQKEDDKKFHILIGATGSVATIKVPLIIDKLF      332
              :   ..*:      *   .            *  :*:::.:***:: .*:  ::.:*

UmHAL3      TY---ANVRVQVIATDNSLHFYDRADIAKLNAASGGDG--DEYTVASLAAENQSASVCGR      1287
ScHal3      EIYGRDRISIQVILTQSATQFFEQRYTKKIIKSSEKLNKMSQYESTPATPVTPTPGQCNM      347
ScCab3      KIYGPEKISIQLIVTKPAEHFLKG------------------------------------      356
                  .: :*:* *. : :* .

UmHAL3      ---AASHVRAHLWTNADEWTSFS-------RIGDPILHIELRRWADMVLIAPCSANTLAK      1337
ScHal3      AQVVELPPHIQLWTDQDEWDAWK-------QRTDPVLHIELRRWADILVVAPLTANTLSK      400
ScCab3      ---LKMSTHVKIWREEDAWVFDAVNKNDTSLSLNLILHHELRKWADIFLIAPLSANTLAK      413
                    : ::* : * *              : :** ***:***:.::** :****:*

UmHAL3      IYGGMCDDLLTSFVRALARDTPKWMFPAMNTLMWENEVTEVHVDALRRR-GWV-VHGPVE      1395
ScHal3      IALGLCDNLLTSVIRAWNPSYPILLAPSMVSSTFNSMMTKKQLQTIKEEMSWVTVFKPSE      460
ScCab3      LANGICNNLLTSVMRDWSPLTPVLIAPAMNTFMYINPMTKKHLTSLVQDYPFIQVLKPVE      473
            :  *:*::****.:*      *  : *:* :  : . :*: :: :: .   :: *  * *

UmHAL3      KML-ACGDMGTGAMVEWTELVQTLVQWAHLVRDESR------------------------      1430
ScHal3      KVMDINGDIGLGGMMDWNEIVNKIVMKLGGYPKNNEEEDDDEDEEEDDDEEEDTEDKNEN      520
ScCab3      KVL-ICGDIGMGGMREWTDIVEIVRRRINEIRKARDEETGDKEQEQEEQEGADN------      526
            *::   **:* *.* :*.::*: :        .

UmHAL3      ----------------------------------------------------- 1430
ScHal3      NNDDDDDDDDDDDDDDD----------DDDDDDDDDEDEDEAETPGIIDKHQ* 562
ScCab3      -EDDDDEDDEEDEEDEEEEEALNETASDESNDEEDEEDEEDVK--TEV*---- 571
```

Supplementary Figure S8. Sequence of the hybrid protein UmHal3_PD_ScCtD as expressed from plasmid pWS93. The tripe HA epitope is underlined and the acidic tail from ScHal3 is denoted in yellow background.

M<u>YPYDVPDYAGYPYDVPDYAGSYPYDVPDYAEF</u>PLPASLRSPYAPLSRPPTCDRPLHIVLASTGSVASVKIPLIVQE

LLTYANVRVQVIATDNSLHFYDRADIAKLNAASGGDGDEYTVASLAAENQSASVCGRAASHVRAHLWTNADEWTSFS

RIGDPILHIELRRWADMVLIAPCSANTLAKIYGGMCDDLLTSFVRALARDTPKWMFPAMNTLMWENEVTEVHVDALR

RRGWVVHGPVEKMLACGDMGTGAMVEWTELVQTLVQWAHLVRDESRPKNNEEEDDDEDEEEDDDEEEDTEDKNENNN

DDDDDDDDDDDDDDDDDDDDDDDDEDEDEAETPGIIDKHQ*
